# Modus Operandi of Medicines, Chemically Considered. No. 2

**Published:** 1850-09

**Authors:** M. M. Rodgers

**Affiliations:** Rochester


(*To be Continued.*)

---

ART. IV.—*Modus Operandi of Medicines, Chemically Considered.* No. 2. By M. M. Rodgers, M. D.

The manner in which medicinal substances produce their curative effects in a pathological condition of any organ or system of organs, and also their pathogenetic effects in a state of health, is very little understood. And although this knowledge may not be indispensable to the successful administration of medicines in the cure of disease, yet in the practice of an art which professes to be founded upon inductions from the exact sciences, it is desirable, if possible, to trace the connection between every effect and its ultimate cause. The explanation given by authors, of the modus operandi of therapeutic agents, falls far short of any thing satisfactory; it is at best, only what relates to their remote effects, and does not reach the ultimate action or final relation which exists between them and the fluids and tissues of the body.

There are three modes, according to authors, in which the general operation of medicines may be explained. 1. It is said, "they produce their effects by actual contact with one or more tissues." But let us go a little farther back, and inquire, how do they act by contact? If we can find what the immediate chemical relations between them and the tissues and fluids are, we shall then have a point from which we can pursue them, step by step, until we arrive at the most remote change produced. When an acid and an alkali are mixed together, effervescence is produced,—and what caused the effervescence? It is caused by the union of the two constituents of the compound. But how was this union produced? By chemical affinity. But again, what causes chemical affinity? Here we must resort to conjecture, and say, perhaps it is caused by cohesion, which is itself caused by a particular internal molecular arrangement,—or, perhaps, by the molecules of each body being in opposite electrical states. But here the explanation ends, and we are still in the dark. And it is true of all investigation, that a limit is set, beyond which we can never pass. We may trace one effect to its legitimate cause, and this cause to some other more remote, which is still but the effect of some cause more deeply hidden, until all beyond is conjecture, and we must link the chain of our reasoning to the Great First Cause.

2. It is said "medicines act by an impulse conveyed by the nerves, through an impression made elsewhere." But here it is only assumed, (not proven,) that the impression is made elsewhere than on the nerves. How do we know whether the primary impression is made upon the nerves or other tissues? The nerves, instead of serving as mere conductors to impressions, may have suffered some lesion, or chemical change, which may increase or diminish their power of generating or conducting any impression whatever. This supposition does not assist the explanation at all.

3. "Medicines act by continuous or contiguous sympathy; or by that which is excited by mere continuity and proximity of parts." Now, to say that a medicine operates by sympathy, is merely to give a name to our ignorance. Sympathy is not a physical or chemical action between particles of *matter*, but a hypothetical term, implying some metaphysical action or condition. No such therapeutical force can be proven to exist in the system: and when a distant organ feels either the pathogenetic or curative effect of a medicine, it must be from a chemical or electrical action upon one or more elements of some tissue or fluid, which is felt along the course of the tissue to the organ in question.

We have now given a synopsis of the modus operandi of the entire materia medica: we may now give briefly the explanations of authors, of a few particular classes of medicines.

*Tonics* produce an augmented action of the circulation, temporary strength, and, finally, fever, when taken in a state of health; but this condition is followed, after a short time, by collapse and debility. In both health and disease, they tend to dry up the secretions and excretions, and thus act as astringents; in this way they arrest night sweating, diarrhœa, and other excessive discharges. In this way a reaction is produced upon the current of the circulating fluid, which causes the tide to set back upon the system, and prevent depletion from morbid action. In this way, also, arterial blood is economized for nutrition, while the vis medicatrix naturæ restores health.

*Febrifuges are* antiperiodics in their action, and, to some extent, are all tonics; they are supposed to terminate periodical diseases by imparting temporary strength and stimulus to the system, which interrupts their paroxysms, while the morbid functions are restored to healthy action by nature herself.

*Nauseating medicines* restrain hemorrhage by causing faintness, which relaxes muscular energy, and thus lessens the force of the circulation, and allows coagula to form at certain points and close the bleeding vessels.

*Purgatives* mostly operate by stimulating the muscular coat of the intestines, and thus increasing peristaltic action. The purgative effect of mineral waters is supposed to depend on the large quantity of water which holds in solution small quantities of mineral salts. The same salts dissolved in any water, has the same effect. They are supposed to act by the stimulus of distention; so that their operation does not depend on the substances they contain, nor on their peculiar combination.

Now these explanations are perhaps correct enough so far as they extend,—but they do not show the ultimate relation between the medicine and any particular substance or tissue of the organism. They give the aggregate of a series of chemical and mechanical processes which had occurred primary to them,—so that they do not explain the specific effect of any medicine or class of medicines.

We shall now consider the manner in which all medicines must be primarily related to the different chemical elements of the body, and attempt to show that there are only two ways in which every article of food, or medicine, whether solid, liquid, or gaseous, must act when taken into the system. It will then be apparent that what we call the specific operation of medicines, is not really any part of their action, but only the manifest consequences of preceding chemical action: that tonics do not directly impart tone to muscular fibre; that cathartics do not directly increase peristaltic action; that emetics do not act by producing nausea; that febrifuges do not cure fever by interrupting the paroxysms, &c. The only two modes in which any substance can act upon the system as a medicine, are by chemical affinity and electricity; mechanical effects often result from these, but are no part of the primary action. These are the only means by which elementary changes take place in bodies, whether organic or inorganic. This will be more apparent when we consider the conditions necessary for the development of either of these forces. Electrical action may be excited between bodies either moist or dry; between gases, liquids, and solids: chemical action can only occur when moisture is present, excepting by high heat, or slow oxydation. When two certain substances, both dry, are brought in contact, electricity may be excited; when two certain substances, one or both moist, are brought in contact, electricity, or chemical affinity, must, one or both, be developed. Whenever this is the case, a change of elements must take place between the two bodies; the old union is broken up, and new ones are formed; so that these elements have different relations to each other, and to all other elements: in the system, a long series of chemical changes may follow this first separation and reunion, and part of these changes may be manifested as the effects of medicines.

One obstacle to our understanding the modus operandi of medicines, is, that the changes which follow their passage into the system, are concealed almost entirely from our view. Another is, that the elementary composition of the fluids and tissues is not constant in quantity or quality. But the greatest difficulty probably consists in the mixed and complicated nature of medicinal substances, and especially those from the vegetable kingdom. Their numerous elements are all compatible in the vegetable during its life; and all so combined as to allow the full development of the organism, the perfect performance of the vital functions, and the consummation of its design. But when vitality ceases, the juices evaporate, volatile matters escape, the organic elements undergo metamorphosis, and new compounds are formed. So that the chemical character is different in the dead plant, and the constituents variable, and their union unstable. In consequence of this weakness of affinity, between the elements of organic bodies, their equilibrium is easily overcome by any disturbing force; so that the chemical character of any vegetable medicine is no index of its operation on the system: we cannot predict what changes it will undergo, and what new compounds will be formed, when it meets with the acids, alkalies, salts, and gases, in its course. Opium, for example, is a complex substance, among the elements of which are, the alkalies, morphia, narcotine, codeine, thebaine, and narceine, besides tannin, extractive and coloring matter, &c. Some of these substances have a strong affinity for others which are held in solution, in unstable combination, by the fluids of the system. So that we must commence with these first changes, in order to trace the operation through all its windings until it leaves the system. And in order to do this, we must know the relative affinities of the elements of the medicine to each other, and to those of the organism; we can then trace the effects of separation and re-union, and the counteracting effects of one upon another, until no farther chemical change takes place.

But from the complex character, feeble union, and wide range of affinities, of all organic compounds, we must consider this almost impracticable. The only way, then, to study the operation of medicine, in accordance with chemical laws, is to use those of the most simple and well known composition,—such as acids, alkalies, salts, oxides, alcohol, ethers, and vegetable proximate principles.

There are several forces besides chemical affinity and electricity, which are supposed to exert some influence on the operation of medicines,—they are, vital principle, animal heat, magnetism, mental action, idiosyncrasy, &c., most of which must be considered only hypothetical.

Galvanic, or electro-galvanic currents, may be developed by the action of free acids, often present in the stomach, upon the mineral elements of salts and oxides: these currents possess electro-positive and electro-negative power, and tend to decompose and revive elementary combinations. They may also cause decomposition in some of the tissues of the body, since their affinities are weak, and they all contain, in their normal state, more or less mineral matter. In this way the action of medicines is modified in some cases to an important extent. Without farther general remark, we will proceed to the consideration of the specific action of some medicinal substances according to our theory.

*Operation of Alcohol.* It is a principle in natural philosophy, that all bodies, in passing from a rarer to a denser state, evolve heat. Alcohol is lighter, and less dense than water; when it is taken into the stomach, it mingles with gastric juice, which is mainly water holding in combination muriatic acid,—this fluid is much heavier than alcohol,—consequently when the union occurs, the dense fluid in becoming rarified, gives off several degrees of latent heat. This accounts for the first calorific effects felt in the stomach. This heat stimulates arterial action by its tendency to expand the blood and other fluids and tissues,—this expansion, while it augments the bulk of the circulating fluid, diminishes the calibre of the vessels, thus making a quicker motion of the blood necessary,—this increased velocity increases the friction between the blood and the sides of the vessels, and consequently increases the heat. Alcohol, by its tendency to coagulate the albumen of the blood, causes in this way obstruction to the circulation, and this is followed by swelling of certain parts from capillary congestion; this capillary congestion reacts upon the larger vessels behind the congested point, causes them to become distended and enlarged, and thus to exert an injurious pressure upon the nerves lying near them,—when this congestion extends to the brain and spinal cord, sensation begins to diminish, motion becomes irregular and involuntary, the senses fail,—and if the congestion continues long, complete apoplexy, stupor, and sometimes death follow. This is intoxication; and when it terminates in resolution, or passes off without any serious injury to the system, all the functions are gradually restored to health. It may be thus explained: part of the alcohol passes from the circulation, with the excretions, while a part is oxydized in the capillaries, to produce animal heat, which has partly in this way been greatly increased. After part of the elements of alcohol have been disposed of in this way, the residue are left in the form of carbon and water, and pass off as effete matter.

Alcohol, then, acts as a *stimulant* upon the vital powers, and particularly on the muscular system, by furnishing to the different tissues, through the arteries, an increased supply of highly oxygenized blood: it acts, afterwards, as a sedative, by producing pressure upon the brain and nerves by congested vessels, and also by deteriorating the quality of the blood. The blood in the congested capillaries, and, finally, of the lungs and whole system, becomes surcharged with carbon, and deficient in oxygen. Thus the vitalizing agent, oxygen, is cut off, and the powerful sedative agent, carbonic acid, furnished in excess. That this is the explanation of the modus operandi of alcohol, is proven by the pathogenetic effects,—by post mortem appearances, by analogy, and by the treatment necessary to cure poisoning by this medicine.

(*To be Continued.*)

ROCHESTER, August, 1850.

